# Magnetic Location of Buried Needles

**Published:** 1915-05

**Authors:** 


**Magnetic Location of Buried Needles.** Geo. H. Monks of Boston, Boston M. & S. Jour., Feb. 25, suggests the following method, used experimentally but not tested clinically: Magnetize the needle by means of a strong electro-magnet placed as near it as possible. Suspend another magnetized needle by a silk thread and use the latter as a divining rod.

